# Supplementary material for: Conventional and Real-Time PCR Targeting blaOXA Genes as Reliable Methods for a Rapid Detection of Carbapenem-Resistant Acinetobacter baumannii Clinical Strains
Source: Antibiotics (Basel). 2022 Mar 28;11(4):455. doi: 10.3390/antibiotics11040455 (PMC9032895; doi:10.3390/antibiotics11040455)
Supplement: Supplementary file 1 [file antibiotics-11-00455-s001.zip › antibiotics-1637020-supplementary.pdf]

Table S1. The origin of *A. baumannii* (*n* = 58) strains used in the present study.

Clinic/Unit	Strains number (%)
Anesthesiology and Intensive Care	40 (69.0%)
Cardiology and Internal Diseases	5 (8.6%)
Liver Surgery and General Surgery	4 (6.9%)
Vascular Surgery and Angiology	4 (6.9%)
Other*	5 (8.6%)

* one strain isolated from each of the following: Cardiac Surgery, Intensive Unit of Cardiac Surgery, Geriatrics, Endocrinology and Rehabilitation Clinics

Table S2. The origin of *A. baumannii* (n = 58) strains used in the present study with respect to clinical specimen type.

Clinical material	Strains number (%)
Bronchoalveolar lavage	26 (44.8%)
Wound swabs	13 (22.4%)
Blood	7 (12.1%)
Urine	3 (5.2)
Vascular catheter	2 (3.4%)
Peritoneal swabs	2 (3.4%)
Other*	5 (8.6%)

* one strain was isolated from each of the following: cerebrospinal fluid, pus, tissue sample, discharge from respiratory tract, granulation tissue

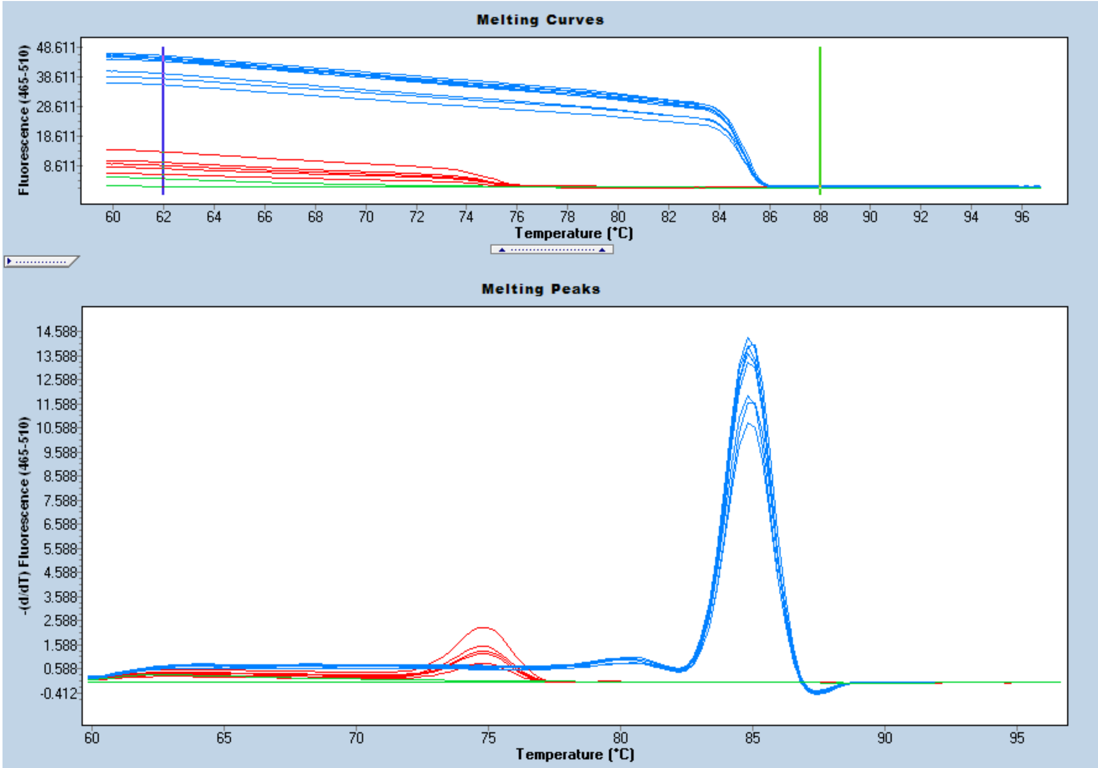

Figure S1. An example of melting curves and melting peaks picture showing specificity of the real-time PCR product for *bla*$_{OXA-23}$ gene (blue lines).
